# The role of parental religiosity in shaping paternal investment: evidence from Bangladesh and India

**DOI:** 10.1098/rspb.2025.1352

**Published:** 2025-08-20

**Authors:** Radim Chvaja, John H. Shaver, Laure Spake, Anushe Hassan, Nurul Alam, Rajesh Kumar Rai, Rebecca Sear, Richard Sosis, Mary Katherine Shenk

**Affiliations:** ^1^ Religion Programme, University of Otago, Dunedin 9016, Otago, New Zealand; ^2^ Faculty of Economics, European Research University, 702 00 Ostrava, Czech Republic; ^3^ Department of Anthropology, Baylor University, Waco, TX 76798-7173, USA; ^4^ Department of Anthropology, Binghamton University, Binghamton, NY 13902-6000, USA; ^5^ Population Health, London School of Hygiene and Tropical Medicine, London WC1E 7HT, UK; ^6^ ICDDRB Public Health Sciences Division, Dhaka 1212, Bangladesh; ^7^ Society for Health and Demographic Surveillance, Suri, West Bengal, India 731101; ^8^ Human Nutrition Unit, Mahidol University, Nakhon Pathom 73170, Thailand; ^9^ Department of Global Health and Population, Harvard University, Boston, MA 02115, USA; ^10^ Centre for Culture and Evolution, Brunel University of London, Uxbridge UB8 3PH, UK; ^11^ Department of Anthropology, University of Connecticut, Storrs, CT 06269-1176, USA; ^12^ Department of Anthropology, Penn State University, University Park, PA 16802, USA

**Keywords:** paternal investment, parental religiosity, India, Bangladesh, allomaternal care

## Abstract

Among humans, paternal investment has been shown to enhance both fertility and offspring survival. While psychological and ecological influences on human paternal investment are relatively well documented, cultural influences remain less well understood. It has been proposed that religion can be an important socio-cultural factor shaping paternal investment. First, religions often instill pro-family values in fathers, potentially increasing their investment. Second, if religions promote pro-family values in mothers, these values may be communicated through religious behaviours, encouraging greater paternal investment. Alternatively, fathers may use maternal religiosity as a strategic cue of maternal pro-family commitment to reduce their own investment, shifting responsibility to mothers. To evaluate these hypotheses, we analyse data from 1238 children under 17 years old across 822 households in India and Bangladesh. Our findings suggest that in India, paternal religiosity is positively associated with fathers’ housework assistance and emotional support to mothers. In Bangladesh, maternal religiosity is positively associated with paternal emotional support to mothers and child provisioning. In both countries, maternal religiosity positively associates with paternal investment among the most religious fathers. These findings indicate that religion plays a complex role in paternal investment, shaped by the interplay of parental religiosity and socio-ecological context.

## 1. Introduction

Paternal investment plays a crucial role in shaping the development and overall well-being of human offspring [1,2]. This involvement can manifest through direct care, such as spending time together, playing, nurturing or supervising a child, as well as through the provision of essential resources like food, shelter, healthcare and education. The quality and quantity of paternal investment have been shown to significantly reduce infant mortality in many contexts [3,4], improve a child’s educational prospects [5,6], speed the social and reproductive careers of children [6,7] and enhance children’s well-being [8,9]. However, there is considerable variation in the extent of paternal involvement across cultures [2,10–13], with many societies practising collaborative childcare, where the father is just one among many carers [14–16].

Biological, psychological and socio-ecological factors, especially relationship stability, can influence levels of paternal investment. For example, unmarried American fathers invest less in their biological children compared with married fathers [17]. Furthermore, paternal investment is positively associated with greater jealous reactions to partner infidelity in 11 diverse cultures, highlighting the link between paternal investment and psychological responses to relationship destabilization [18]. Additionally, fathers in polygynous rural Senegal invest more in children who closely resemble them, suggesting that kin recognition mechanisms play a role in paternal investment [19]. These findings suggest that fathers may use psychological cues to estimate the likelihood that a child is biologically theirs and adjust their investment accordingly [10], though exceptions exist [20]. Supporting the role of relationship quality, longitudinal research in the USA has found that greater marital satisfaction—assessed through both partners’ reports—is one of the strongest predictors of paternal involvement, including caregiving, playfulness and emotional engagement [21]. Similarly, Belsky’s [22] process model highlights the marital relationship as the primary context for parental support, demonstrating that emotionally supportive partnerships enhance fathers’ investment by improving psychological well-being and reducing their own stress. In summary, perceived instability in the relationship, often influenced by infidelity and other cues that challenge misattributed paternity, may reduce paternal investment, which can lead to poorer outcomes for children.

Recent developments in partner choice and sexual selection models of religion [23–27] suggest religion as a potential mechanism regulating paternal investment. Many religious texts reinforce sexual fidelity and pro-family values through commandments, parables and moral teachings—for instance, the *Quran* emphasizes maternal responsibility towards children and paternal responsibility towards the mother (e.g. Surah Al-Baqarah 2:233: *Mothers may breastfeed their children two complete years for whoever wishes to complete the nursing. Upon the father is the mothers' provision and their clothing according to what is acceptable*). Similarly, Hindu scriptures stress marital fidelity as a core duty, as stated in the Manusmriti (9.1): *Considering that the highest duty of all castes, even weak husbands (must) strive to guard their wives.* Empirical research suggests that religious believers live according to these demands, namely, they engage in lower rates of extramarital intercourse and are less likely to divorce [28–33]. As a result, paternal religiosity may enhance paternal investment by fostering pro-family values and behaviours among fathers [34,35]. Additionally, maternal religiosity may serve as a signal of fidelity and family commitment, potentially encouraging greater paternal investment in her children [23,36].

However, an alternative explanation is that religious fathers, owing to conservative beliefs, may adhere to traditional gender roles, expecting women to be primarily responsible for childcare and household duties [37]. Consequently, religious fathers might invest less in their children [36], believing it is the mother’s role. Likewise, if fathers perceive maternal religiosity as a signal of strong commitment to childcare and household responsibilities, they may reduce their own investment, reallocating their time and energy elsewhere, with the confidence that their spouse will reliably engage in childcare. If correct, maternal and paternal religiosity should be negatively associated with paternal investment.

These theoretical arguments lead to divergent hypotheses about the interplay between maternal and paternal religiosity. If both parents’ religiosity leads to conservative gender norms—namely that women should take care of the household and children, paternal religiosity should strengthen the negative association between maternal religiosity and paternal investment. Conversely, if religion increases pro-family commitment in both parents, paternal religiosity should amplify the positive association between maternal religiosity and paternal investment.

The argument about the signalling value of mothers’ religiosity assumes that the signal is visible to the father. If mothers use religion as a signal of pro-family values and fidelity to fathers, then only private religious behaviours—those observable by the husband—should be associated (either positively or negatively) with paternal investment. This is because private religious behaviours occur within the shared domestic space. By contrast, public religious behaviours, which are, in some Hindu and Muslim communities such as those studied in this research, performed primarily in the presence of other women, and less in the presence of husbands, should not be associated with paternal investment.

Research on the relationship between parental religiosity and paternal investment remains scarce. A recent study found that in the UK, there was no association between maternal religiosity and direct childcare from a mother’s partner, but a positive association between maternal religiosity and a partner’s involvement in household responsibilities; maternal religiosity in the USA exhibited a negative association between maternal religiosity and these two measures of a partner’s investment [36], supporting the idea that fathers may use maternal religiosity as a strategic cue to reduce their own investment in some contexts and increase it in other contexts. Another study conducted among a Muslim population in The Gambia found no evidence of an association between maternal religiosity, maternal veiling and paternal investment, measured as direct physical care and economic provisioning towards a focal child [38]. This suggests that religiosity may not always function as a direct signal to husbands, and that socio-ecological factors, such as fathers spending significant time outside the home and not directly observing maternal religious behaviour, may play a role. Overall, no clear conclusions can be drawn about the relationship between maternal religiosity and paternal investment from the existing literature. Regarding paternal religiosity, a handful of studies indicate that religious fathers tend to invest more time in their children and support the mother. However, this evidence comes predominantly from US samples, and existing measures of paternal investment do not fully capture the range of tasks fathers may perform [34,35].

This study aims to examine the association between maternal and paternal religiosity and paternal investment in a sample of Muslim and Hindu parents and children from rural India and Bangladesh. Both communities are religiously homogeneous (within households) and highly devout, reducing the likelihood of misinterpretation, such as non-religious fathers failing to recognize maternal religiosity [39,40]. Our study makes several key contributions: We use detailed measures of paternal investment, including two composite measures of childcare and two single-item measures of social support to mothers. We further incorporate ethnographically rich measures of maternal private and public religiosity. By including paternal religiosity in our models, we rule out the possibility that any observed relationship between maternal religiosity and paternal investment is merely due to assortative mating, where highly religious men marry highly religious women, who both simultaneously invest more in their children. By examining fathers’ evaluations of maternal religiosity, we explore whether fathers are aware of, and responsive to, maternal religiosity in ways that influence their investment.

Specifically, we test the following hypotheses:

H1.1: Paternal religiosity is positively associated with paternal investment.
H1.2: Maternal private religiosity is positively associated with paternal investment.
H1.3: The slope of interaction between maternal private religiosity and paternal investment is positive.
H2.1: Paternal religiosity is negatively associated with paternal investment.
H2.2: Maternal private religiosity is negatively associated with paternal investment.
H2.3: The slope of interaction between maternal private religiosity and paternal investment is negative.
H3: Maternal public religiosity is not associated with paternal investment.
H4: (Exploratory): If maternal religiosity (private or public) is associated with paternal investment (including interactions with paternal religiosity), we test whether this relationship is mediated by fathers’ perceptions of maternal religiosity.

## 2. Material and methods

### (a) Data

Our data come from rural communities in Bangladesh and India. Surveys asked mothers about household composition, reproductive histories, religiosity, support networks, education and investment in up to two focal children. Moreover, for a substantial proportion of mothers (78.5%), additional data on religion and social support were also collected from their spouses. The probability of having data from the spouse was not influenced by religious factors but was instead determined by logistical constraints, primarily the high rate of male labour migration in the region (particularly Bangladesh), which made many husbands unavailable for interview, as well as challenges related to interviewing husbands at their jobs or in their homes during the evening within the timeframe of the study.

Samples were drawn from cohorts at two field sites run by local Health and Demographic Surveillance Systems (HDSS), one in Matlab, Bangladesh [41] and the other in Birbhum, West Bengal, India [42,43]. The criterion for mothers’ participation was that they had at least one child and were between 25 and 60 years old. Additionally, we aimed to balance the sample by including an equal number of mothers with all children under 17 years old and mothers with at least one child aged 17 or older. Women were drawn at random (given the selection criteria) from population census data at both sites and interviewed in their homes. Data collection was conducted by trained local research assistants using Open Data Kit [44] between March and August 2022, in collaboration with each HDSS, and overseen by one of the co-authors of this study.

The original sample sizes were 1766 focal children (from 1135 households) in India and 1785 focal children (from 1003 households) in Bangladesh. Several selection criteria were applied for this analysis. First, we included only children under 17 years old. Second, we only included children whose father was also interviewed. Third, only children residing in the same household with both parents were included, as fathers' abilities to invest in their children are reduced if they live elsewhere. Step 2, and partially step 3, could introduce a bias towards omitting families where fathers are labour migrants who could not be interviewed or who reported not currently living with the child. We speculated that this may result in wealthier families being omitted in steps 2 and 3. However, our data exploration suggests that this is not the case as the omitted households were not significantly less or more wealthy than households passing the criteria to step 3 (see electronic supplementary material, table S4).

**Figure 1.**
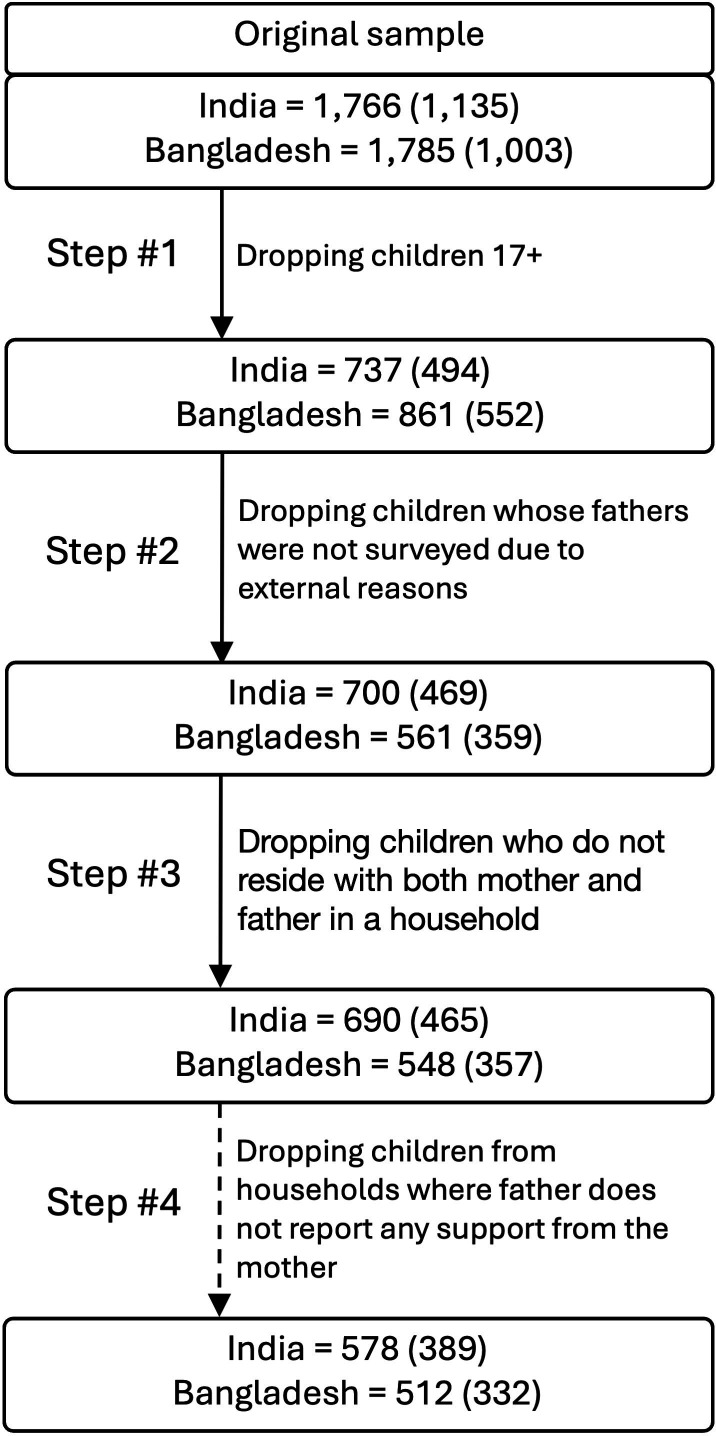
Flowchart illustrating the selection process of focal children. Numbers represent the number of children, with the number of households in parentheses. The dashed line in step 4 indicates a subsample used exclusively for exploratory analyses of paternal perceptions of maternal religiosity. Fathers were asked to list individuals who supported them in six specific domains: assistance with housework, emotional support, financial help, job searches, help during illness and food sharing. Religiosity ratings were only collected for those individuals the fathers listed.

Finally, for analyses involving fathers' perceptions of maternal religiosity, only fathers who reported the mother among people who helped the father with certain tasks (explained in detail in §2c) could be included as only then was the father asked about his perceptions of his wife’s religiosity. We acknowledge that this introduces a selection bias, and thus, results (test of H4) from these analyses should be considered exploratory and interpreted with caution. Figure 1 illustrates the selection process for focal children, while table 1 presents basic characteristics of the final sample after the third selection step.

**Table 1.** Sample description.

	India			Bangladesh		
	*mean* (s.d.)	min.	max.	*mean *(s.d.)	min.	max.
*mother*						
total * number*	465			357		
Muslim	0.54 (0.50)			0.49 (0.50)		
private religiosity	4.40 (1.59)	0	9	6.13 (1.70)	2	11
public religiosity	2.36 (6.40)	0	56	1.21 (2.16)	0	17
age	35.76 (6.63)	22.42	57.00	36.65 (7.16)	24.58	54.67
living children	2.37 (1.01)	1	7	2.33 (0.82)	1	7
emotional support received from father	0.70 (1.38)	0	4	2.12 (1.78)	0	4
housework assistance received from father	1.74 (1.80)	0	4	2.89 (1.62)	0	4
*father* ^a^						
Muslim	0.53 (0.50)			0.49 (0.50)		
perceived maternal religiosity	2.73 (0.76)	1	5	2.87 (0.77)	1	5
education	1.34 (0.91)	0	3	1.38 (0.83)	0	3
age	42.11 (8.00)	26.00	81.33	44.93 (8.02)	25.08	78.42
*household*						
wealth ($US)	54 850 (107 438)	6 439	1 510 166	14 224 (13 919)	4578	120 349
reversed urbanization (minutes)	29.71 (10.65)	9.89	71.67	20.11 (7.05)	3.44	52.03
*focal children*						
total n*umber*	690			550		
boys	0.50 (0.50)			0.50 (0.50)		
child’s age	10.97 (4.19)	1.25	17.08	9.33 (4.31)	1.25	16.67
direct care	0.45 (0.82)	0	1	0.34 (0.22)	0	1
provisioning	0.34 (0.52)	0	1	0.50 (0.26)	0	0.925

^a^ We do not display latent score of paternal religiosity, which is standardized by design of its construction (*Mean* = 0, s.d. = 1). Reversed urbanization is expressed as time needed to travel to urban institutions.

### (b) Ethnographic background and data collection

#### (i) Matlab, Bangladesh

This area spans approximately 184 km², with a population density of around 1250 people per km² (as of 2014). Located 55 km from Dhaka, the climate is subtropical, with regular floods. The area is experiencing rapid urbanization owing to migration from rural areas. It currently encompasses around 142 villages in seven administrative blocks, with a population of *ca* 230 000 residents. The ethnic composition is predominantly Bengali, with agriculture, fishing and trade serving as the primary means of subsistence [41]. Approximately 89% of the local population is Muslim, while 11% is Hindu [45]. The HDSS in this area is managed by the International Centre for Diarrhoeal Disease Research, Bangladesh, established in 1966 [41].

#### (ii) Birbhum, India

This area covers roughly 4545 km² with a population density of 771 people per km² (as of 2015) and is located in the state of West Bengal. The climate is subtropical, with regular rainfall. Between 2001 and 2011, the area experienced rapid population growth. It now includes around 350 villages in seven administrative blocks, with a population of *ca *3.5 million residents [42]. The population is predominantly Bengali, with agriculture as the main subsistence activity. In 2011, approximately 60% of the population were Hindu, while 35% were Muslim [46]. The HDSS in Birbhum is managed by the Society for Health and Demographic Surveillance, established in 2008, with funding from the Department of Health and Family Welfare, Government of West Bengal [42].

In both Birbhum and Matlab, most mothers live in extended-family-based neighbourhoods (*bari*) and spend most of their time managing household tasks, often interacting with kin and other women. While religious institutions like mosques (*masjid*) and temples (*mandir*) are present, religious activities at home and within the *bari* are generally more important for local women than institutional religious practices. Muslim women typically do not pray at local mosques, but instead at home—and while Hindu women do perform worship ritual (or *puja*) at local shrines at home and in the neighbourhood, it is less common for them to worship at formal temples unless accompanied by other family members.

### (c) Variables

#### (i) Maternal religiosity

We used two independent indicators of maternal religious behaviour—private and public. Private religious behaviour, such as prayers, recitations and personal rituals performed at home, is likely to be directly observed by fathers because they share the same household. By contrast, public religious behaviours, such as collective prayers and religious discussions, are less consistently observed by fathers, as women often perform these activities with other women in female-only spaces at times and places where men are generally absent. We utilized ethnographically sensitive measures that account for religious differences, as Muslim and Hindu women follow different norms surrounding religious practices and rituals. We used a method designed to capture the diversity of religious behavioural expressions, rather than assuming a unidimensional or latent structure. Instead of generating a latent score from highly correlated items, we summed different practices, acknowledging that individuals may engage in varying forms of religious behaviour. This approach reflects the idea that participation in one type of activity may limit engagement in others owing to constraints of time, energy or access. Additionally, certain religious practices may be more or less available depending on local norms, spatial context or life circumstances. By using this additive strategy, we aimed to preserve meaningful variation and respect the heterogeneity of religious expression across individuals and communities.

To create an index of private religiosity, we asked both Muslim and Hindu women whether they engaged in the following activities: (1) reading or reciting religious texts, (2) praying, (3) visiting religious websites or YouTube channels, (4) reading books about religion, (5) watching religious programmes on TV or listening to the radio, and (6) other similar activities. Additional religion-specific questions were asked to account for the distinct practices of each religion. Muslim women were asked whether they (7) performed all five obligatory prayers daily, (8) fasted during Ramadan, (9) learned to recite the *Quran* during childhood, and (10) currently recite the *Quran*, either from memory or directly from the text. Hindu women were asked about practices such as (7) watering a basil plant, (8) reciting mantras from the (9) *Rudraksh* and (9) *Geeta*, (11) participating in ceremonial fasting, and performing religious rituals of (12) prayer and (13) worship (*puja*). We summed the positive responses to both shared and unique activities, creating an index with a potential range of 0−10 for Muslim mothers and 0−13 for Hindu mothers. The index was standardized within each religion and location to estimate within-group effects.

To create an index of public religiosity, we asked women how many times in the past four weeks they had attended a temple or mosque for prayers or participated in religious discussions, and how often they attended any religious event or gathering outside of temple or mosque, such as in their neighbourhood or a nearby neighbourhood. We summed these numbers and standardized them within each religion–location group.

#### (ii) Paternal religiosity

Rather than focusing on fathers’ public or private religiosity, we concentrated on their subjectively evaluated religiosity because we do not analyse others’ receptiveness of fathers’ religious behaviour but rather the motivational role of paternal religiosity in their investment. We used three items asking fathers how pious they are, and how religious they are as compared with their family and community members. The resulting scales had sufficient internal reliability (see electronic supplementary material, table S1 for details).

#### (iii) Paternal investment

We followed the established literature [18,20,38] to construct two indices of paternal investment: one for direct care and one for provisioning. Overall, factor analysis supported this structure, though with minor differences between countries.

Direct care is based on activities that require direct contact with the focal child. Depending on the age of the focal child, mothers were asked who, in the past 2 weeks, washed the focal child (i), cooked for them (ii), supervised them (iii) and disciplined them (iv; age: 0−4); cooked (i), supervised (ii), helped with schoolwork (iii), and disciplined (iv; age: 5−16). Next, we asked how often each of the people performed each of the tasks (multiple times per day, once a day, a few times per week, once a week, once in 2 weeks, never).

Provisioning is based upon resources that fathers invest in children while not necessarily being physically around them. Mothers were asked, again depending on the age of the focal child, who in the past three months paid for child’s medical expenses (i), gave them food, money or gifts (ii), and took them shopping (iii, age: 0−4); paid for the child’s medical expenses (i), educational expenses (ii), gave them food, money or gifts (ii), and took them shopping (iv, age: 5−16), and how often the person performed each of the tasks in the past three months (more than five times, four to five times, two to three times, once, never).

For each age group in both countries, we ran factor analysis for ordinal variables using the *psych* package [47], which suggested clear two-factor solution. However, the shopping item loaded on the direct care factor in Bangladesh but on the provisioning factor in India. Since it makes sense that taking children shopping includes physically spending time with them, we constructed latent scores, reflecting that shopping loaded on direct care in Bangladesh (details in electronic supplementary material, table S2). To construct latent scores, we first rescaled variables to a range of 0−1, assigning a value of 1 to the highest possible frequency (e.g. ‘multiple times per day’ for direct care and ‘more than five times’ for provisioning) and 0 to the lowest (‘never’), regardless of whether those extremes were observed in the data. Next, we averaged the respective variables and then assigned 0 to those children whose father was not listed among allomothers.

In addition to the two variables assessing paternal direct engagement and provisioning of their children, we followed Chvaja *et al*. [40] to include two broader evaluations of paternal investment, specifically the frequency of assistance with housework tasks to the mother and emotional support to the mother. To obtain a measure of housework assistance, we asked mothers to list people who helped them with work in the past year (e.g. housework, garden work or other). Next, we asked how often the person provided housework assistance (1 = *less than monthly*, 2 = *monthly*, 3 = *weekly*, and 4 = *daily*). In the case of emotional support, mothers listed people who had provided them with emotional support in the last year. For each of the selected supporters, we asked how intense the emotional support was (1 = *very little*/*not intensively at all*, 2 = *a little but not intensively*, 3 = *intensively,* or 4 = *very intensively*).

#### (iv) Paternal perception of maternal religiosity

An important measure in our study is how fathers perceive the religiosity of their spouses. Fathers were asked a battery of questions on the social support they receive from others. If a father named his spouse as a source or target of support in any domain—such as emotional support, housework assistance, financial support, providing food, help during illness or help with job seeking—he was subsequently asked about basic characteristics of his spouse, including how religious she was compared with most people in their village. Responses were recorded on a 5-point scale (1* = much less*, 2* = a little less*, 3* = about the same*, 4* = a little more*, 5* = much more*).

#### (v) Control variables

We controlled for variables that could represent confounders in the hypothesized relationship between maternal religiosity and paternal investment: paternal education and age, household wealth (see electronic supplementary material, §S3), household urbanization (see electronic supplementary material, §S5), and number of living biological children reported by the woman. We also controlled for maternal religion (Muslim or Hindu), child’s sex and age to increase the precision of the estimates in models where the child is the unit of observation.

#### (vi) Analytical approach

All analyses were conducted using R [48]. To model direct care and provisioning, we used multilevel *ordinary least squares* (OLS) regressions with random intercepts for household as some households had more than one child in the study (package *glmmTMB*; [49]). To model emotional support and housework assistance, clearly ordered categorical variables, we used *cumulative link models* (CLMs) that estimate the log-odds of moving into a higher category (package* ordinal*; [50]). We built two models for each out of four outcome variables (two childcare variables, two social support variables), one for India and one for Bangladesh. All predictors, outcomes and control variables were standardized before entering in the models. Binary variables (child’s sex and woman’s religion) were centred around the mean (−0.5 vs 0.5), meaning that estimates are reported for population averages. Missing values (5.47% missing in paternal religiosity responses and 0.97% in maternal private religiosity responses) were imputed with country medians. Model equations are accessbile from electronic supplementary material, §S2 (eqns 1–3).

## 3. Results

Table 1 suggests that, overall, fathers in Bangladesh tend to support their spouses more and are more engaged in childcare than fathers in India. The correlation across the whole sample between direct care and provisioning was *r* = 0.64 (*p* < 0.001), suggesting a shared tendency to invest in children rather than specialization in one type of care. With social support, the correlation between the two measures was lower (*r* = 0.27, *p* < 0.001), suggesting that emotional support and housework assistance are two distinct forms of social support. Finally, the correlation between the two aspects of maternal religiosity were very weakly correlated (*r* = 0.11, *p* < 0.001), suggesting that these two measures capture two distinct modes of religiosity, allowing us to include them both in one model without the potential influence of multicollinearity (highest variance inflation factor in our models was 1.46, suggesting no multicollinearity issues).

**Figure 2.**
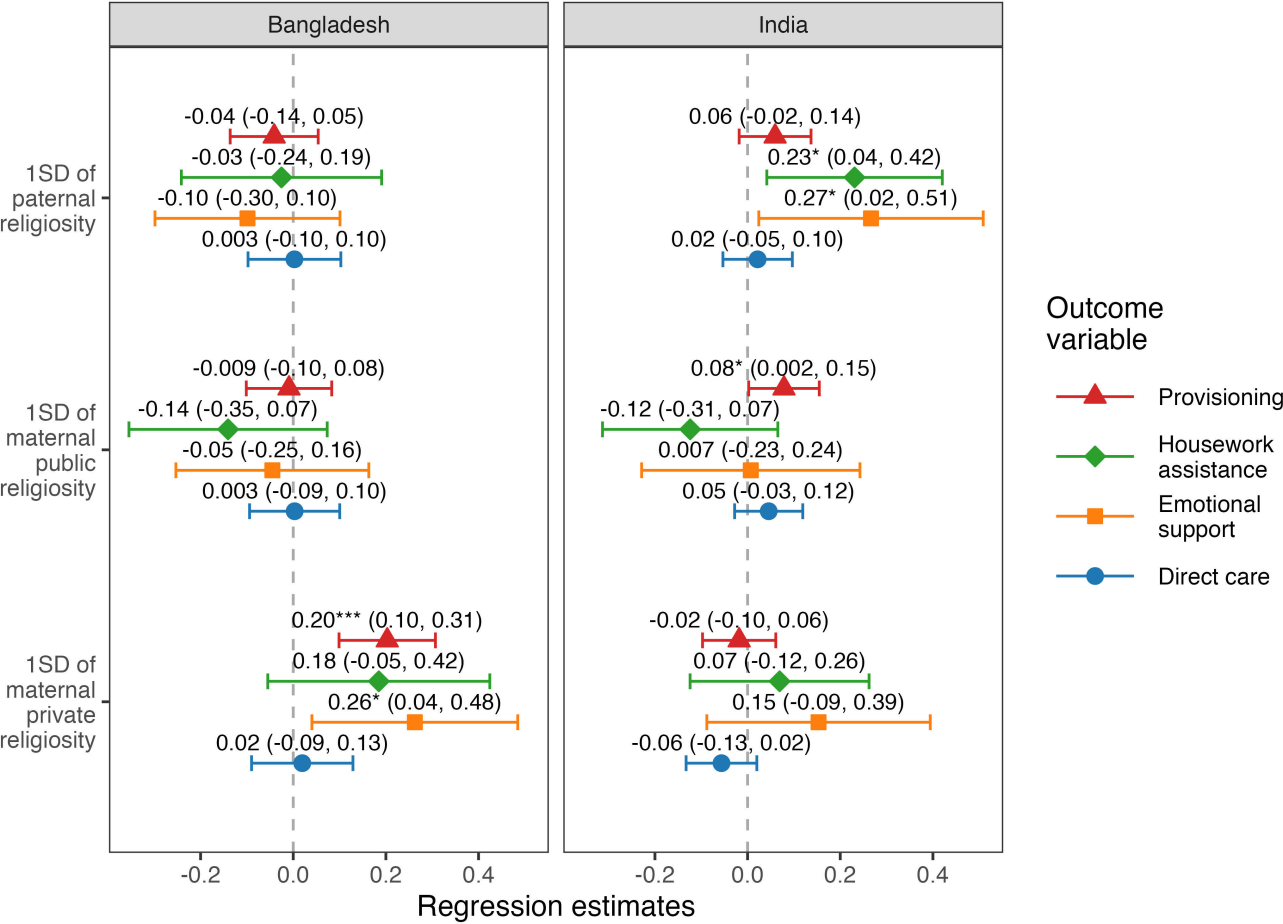
The ordinary least squares (provisioning and direct care) and cumulative link model (housework assistance and emotional support) estimates from the main models. The points and error bars represent estimates and 95% confidence intervals of the effects of maternal and paternal religiosity on the four measures of paternal investment (outcome variables). SD, standard deviation. * p < 0.05, ** p < 0.01, *** p < 0.001

Models displayed in figure 2 (all models in the Results are detailed in electronic supplementary material, tables S6–S8) found support for the hypothesis that more religious fathers tend to invest more in their children (H1.1). In India, we observed a positive association between paternal religiosity and housework assistance and emotional support by fathers. No support was found for the hypothesis that paternal religiosity negatively correlates with paternal investment (H2.1). We found support for the hypothesis that maternal private religiosity is positively associated with paternal investment, namely with child provisioning and emotional support (H1.2), and no support for the hypothesis that maternal private religiosity and paternal investment are negatively associated (H2.2) in Bangladesh. In India, however, none of the investment measures was associated with private maternal religiosity, suggesting a lack of support for H1.1 or H2.1 in that context. Testing whether public maternal religiosity associates with paternal investment (H3), we found a weak positive association on the edge of statistical significance between maternal public religiosity and paternal provisioning in India (we predicted no association).

**Figure 3.**
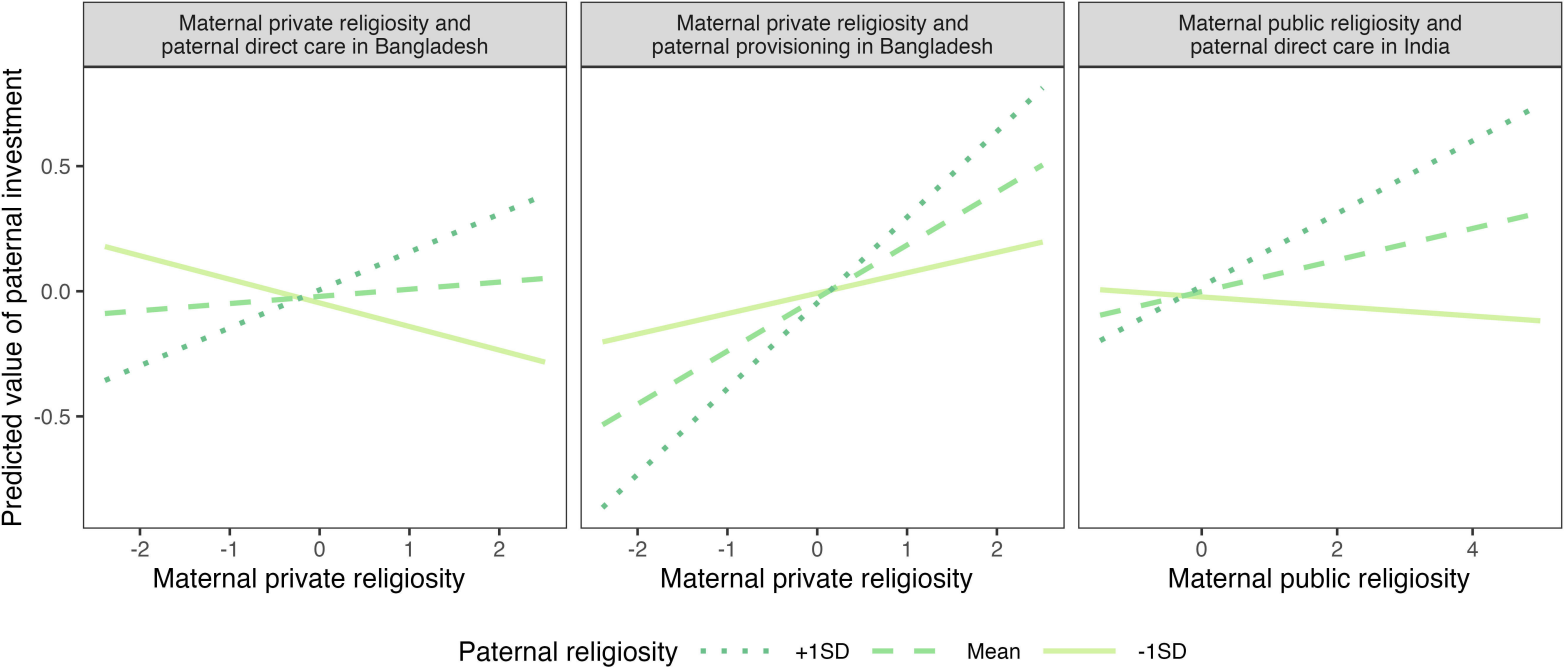
Predicted values of paternal childcare in Bangladesh based on the fully controlled ordinary least squares regression model. Note that all variables are standardized.

In terms of the hypotheses predicting that an association between maternal religiosity and paternal investment should be moderated by paternal religiosity (H1.3 and H2.3), we found that maternal private religiosity positively interacted with paternal religiosity in Bangladesh to predict paternal direct care (*Β* = 0.12, 95% CI = [0.03, 0.22], *p* = 0.014) and provisioning (*Β* = 0.13, 95% CI = [0.04, 0.22], *p* = 0.005). We also found a positive interaction between maternal public religiosity and paternal religiosity in predicting direct paternal childcare in India (*Β* = 0.08, 95% CI = [0.01, 0.16], *p* = 0.031). Figure 3 displays all the significant interactions. No other interaction was found.

Since we found associations between maternal private religiosity and paternal investment in Bangladesh and between maternal public religiosity and paternal provisioning in India, we explored a possibility that these may be mediated through increased perception of maternal religiosity by fathers (H4). We began with exploring whether maternal private and public religiosity was positively associated with a perception of maternal religiosity by fathers, but we found no evidence for such a relationship (India, private religiosity: log-odds = 0.06, 95% CI = [−0.19, 0.31], *p* = 0.625; India, public religiosity: log-odds = −0.02, 95% CI = [−0.24, 0.20], *p* = 0.864; Bangladesh, private religiosity: log-odds = 0.21, 95% CI = [−0.03, 0.44], *p* = 0.086; Bangladesh, public religiosity: log-odds = 0.07, 95% CI = [−0.13, 0.28], *p* = 0.481). Therefore, we did not proceed with a mediation analysis. Interestingly, we found that in both India (log-odds = 2.04, 95% CI = [1.74, 2.35], *p* < 0.001) and Bangladesh (log-odds = 0.51, 95% CI = [0.28, 0.73], *p* < 0.001), more religious fathers perceived their spouses as being more religious.

## 4. Discussion

We studied the complex role of parental religiosity in paternal investment. Our key finding is that fathers do not appear to strategically reduce their involvement based on the religiosity of the mother. On the contrary, fathers tend to invest more when mothers engage in visible religious activities, and this effect is especially pronounced among highly religious fathers. That said, the results were not uniform across all measures: significant associations emerged only for certain dimensions of maternal religiosity and specific types of paternal investment, which differed in India and Bangladesh. Overall, the findings suggest that parental religiosity may be linked to greater paternal involvement—either through a direct motivational effect or indirectly, by signalling a pro-family orientation on the part of mothers [23–25].

The overall weak support for the hypothesis that maternal religiosity associates with paternal investment may be due to the relatively low risk of extramarital affairs in the studied populations—approximately 90% of the women in our sample reported being housewives. This limited exposure to unrelated men outside the household, such as in the workplace, may reduce the signalling relevance of religiosity in these contexts. The observed differences between India and Bangladesh suggest that local socio-ecological factors may play an important role in shaping the relationship between religiosity and paternal investment. One plausible explanation relates to differences in paternal absence and the opportunities it creates. In our data, Indian fathers are more present in the household—only 12.04% travel away daily—compared with 36.41% of Bangladeshi fathers. Greater absence in Bangladesh may create more opportunities for extramarital affairs for women, making maternal religious signalling a more important strategy to signal chastity. This could explain why maternal religiosity has stronger effects on paternal investment in Bangladesh. 

The cooperative role of religious signals [51–54] is particularly pronounced when both fathers and mothers are highly religious, meaning both the signaller and the signal receiver share the cultural system of beliefs that motivates the signals [39,40,55]. In fact, religious signals transmitted by mothers in Bangladesh were even detrimental for paternal provisioning if their spouses were not religious. When fathers were more religious, the association between maternal religiosity and paternal investment strengthened, becoming strongly positive among the most religious fathers. This suggests that the overlap between parental belief systems may be necessary for maintaining parental cooperation. Alternatively, this effect of homogamy in couples does not need to be unique to religion but also applies to any trait including political ideologies, occupation or age [56].

Following the idea that families where both parents are highly religious exhibit higher levels of intra-parental cooperation, our exploratory findings suggest that more religious fathers perceive their spouses as being more religious, even after controlling for the mothers’ self-reported religious practice. This indicates that fathers tend to project their own religiosity onto their spouses. Such psychological processes are well documented and suggest that people tend to project their own qualities onto others [57], particularly ingroup members [58]. This automatic cognitive process likely facilitates cooperation by increasing perceived homogeneity and projecting cooperative intentions onto others [58,59].

The discrepancy between the effects of public and private maternal religiosity is expected by signalling theory. In the study communities, private religiosity involves religious activities performed at home, where a woman’s spouse can observe her behaviour. Public religious activities, on the other hand, are typically conducted with other women, outside the presence of men. Therefore, in the context of signalling to a spouse, private religiosity is a more effective signal. This strengthens the causal interpretation of the association between maternal religiosity and paternal investment. If the association was due to unmeasured confounding, public religiosity would also need to be associated with paternal investment. A recent study using our data from Bangladesh suggests that maternal private religiosity is positively associated with the frequency of alloparental childcare provided by household members, but not by others [60]. However, the same study found no association between maternal public religiosity and alloparental care provided by anyone. Interestingly, fathers’ public religiosity was positively associated with childcare provided by alloparents across all categories [60]. These findings suggest that different types of religious behaviour may serve as signalling modalities tailored to different genders, needs and contexts [61,62].

When interpreted together with findings by Samore *et al*. [60], our study highlights that paternal investment, even when studied in relation to cultural factors like religion, is embedded within a larger network of allomaternal and maternal care. Focusing solely on paternal investment, without considering the contributions of other carers, may lead to biased conclusions about the role of religion in specific communities. This is particularly important when religion is conceptualized as a complex adaptive system [63,64] comprising multiple elements that interact to provide cultural solutions to local socio-ecological challenges [65]. A function of religion that is pronounced in one community may be missing in another community, where religion may solve a different problem. Studies to date suggest that religion’s effects on paternal investment are variable across cultures, but religions’ effects on investment by non-father kin are more stable. For example, Shaver *et al.* [38] concluded that maternal religiosity in The Gambia does not increase paternal investment (we re-ran their analyses with private maternal religiosity instead of overall maternal religiosity score and found no change in results; see electronic supplementary material, §S3) but strengthens cooperative childrearing among matrilateral and affinal kin, whose presence may be more critical for children in that socio-ecological context [66]. A similar pattern was observed by Spake *et al*. [36] in their study of UK and US households: More religious mothers received more childcare and household assistance from non-partner kin in both countries but not from partners (with the exception of household help in the UK).

In our analyses, we found no link between maternal or paternal religiosity and paternal direct care (save for the most religious fathers). Previous research suggests that fathers often prefer non-substitutable investments [7], such as provisioning, rather than direct care, which is typically provided by mothers, older siblings and other female relatives [67–74]. In line with this reasoning, fathers in the studied communities are primarily breadwinners, spending significant time acquiring resources for the family, while physical caregiving is often the responsibility of mothers and other female kin [75,76]. Paternal provisioning, therefore, is the crucial factor that may determine fitness consequences for both the mother and father, and strategies to improve paternal provisioning may be especially effective under such circumstances. Previous research indicates that housework assistance and emotional support may alleviate mothers’ domestic workload and reduce stress, enabling them to focus more on subsequent reproduction [77–79]. Maternal religiosity may, therefore, ultimately increase both parents’ inclusive fitness.

While our findings partially support the hypothesis that maternal religiosity is positively associated with paternal investment, they do not support our additional exploratory hypothesis, that fathers consciously obtain information about maternal religiosity from mothers’ behaviour and use this information to adjust their child investment. This is because fathers do not rate their spouse’s religiosity based upon maternal religious practice. This could reflect our poor measurement of perceived religiosity (one ordinal item) or indicate that fathers do not consciously reflect their spouse’s religious behaviour, or it may imply that the signalled quality is not general religiosity but a specific religious worldview related to pro-family values and fidelity. Future research could address this possibility by measuring fathers’ perceptions of maternal fidelity. The question would then be whether maternal religiosity affects fathers’ perception of their wives' fidelity, which in turn affects paternal investment.

Several limitations of our study should be acknowledged. A key limitation is our reliance on self-reports of religiosity, which may be subject to social desirability bias or individual misrepresentation [80]. This is particularly relevant for private religious practices, which are less observable and may be over- or under-reported depending on cultural expectations. While public religious behaviours, such as mosque or temple attendance, are more easily verifiable by a team of field researchers, private religiosity poses a greater measurement challenge. Future studies could improve accuracy by incorporating reports from other household members—such as children or spouses—to triangulate responses and gain a more comprehensive understanding of religious engagement, reducing bias introduced by self-assessments. Beyond measurement concerns, our study remains limited in its ability to establish causal relationships. While we accounted for key confounders, longitudinal or experimental designs would be necessary to determine the precise direction of effects. Furthermore, our findings are context-specific, drawing from rural populations in Bangladesh and India. It remains unclear whether similar patterns would emerge in more urbanized settings or among communities where religious expression and risk of extramarital affairs differ.

## 5. Conclusion

Over the decades, studies have accumulated evidence showing that childrearing is a cooperative endeavour involving more than just the mother and the father. In some communities, fathers invest less in their children than other family members, such as grandmothers [74]. However, paternal provisioning, along with alloparental investments in general, has likely played a crucial role in human evolution [1,2,71,81] by enhancing fertility, survivorship and the further development of offspring [4,6]. Therefore, securing fathers' involvement could be evolutionarily beneficial for mothers, and various strategies to achieve this are likely observable in communities worldwide. We found evidence that religion may offer one such strategy in India and Bangladesh. Our interpretations align with two major evolutionary theories of religion. The first considers religion a communicative platform [54,82,83]. The second theory understands religion in terms of sexual selection [84,85], accounting for the differential reproductive costs for males and females, and the associated parental investment [25,26,86]. In light of these evolutionary frameworks, our findings suggest that religion may serve as a culturally adaptive solution to the problem of parental cooperation.

## Data Availability

Data and R script necessary to replicate findings and electronic supplement material at [87]. Supplementary material is available online [88].
